# Confirmation that somatic mutations of beta‐2 microglobulin correlate with a lack of recurrence in a subset of stage II mismatch repair deficient colorectal cancers from the QUASAR trial

**DOI:** 10.1111/his.13895

**Published:** 2019-07-05

**Authors:** Paul Barrow, Susan D Richman, Andrew J Wallace, Kelly Handley, Gordon G A Hutchins, David Kerr, Laura Magill, D Gareth Evans, Richard Gray, Phil Quirke, James Hill

**Affiliations:** ^1^ Department of Surgery, Manchester Royal Infirmary Central Manchester University Hospitals NHS Trust Manchester UK; ^2^ Department of Pathology and Tumour Biology, Leeds Institute of Cancer and Pathology St James’ University Hospital Leeds UK; ^3^ Manchester Centre for Genomic Medicine St. Mary’s Hospital, Manchester University Hospitals NHS Trust Manchester UK; ^4^ Birmingham Clinical Trials Unit University of Birmingham Birmingham UK; ^5^ Cancer Medicine University of Oxford Oxford UK; ^6^ University of Oxford Oxford UK

**Keywords:** beta2‐microglobulin (B2M), colorectal cancer, QUASAR, dMMR, pMMR, mismatch‐repair

## Abstract

**Aims:**

Beta2‐microglobulin (B2M) forms part of the HLA class I complex and plays a role in metastatic biology. *B2M* mutations occur frequently in mismatch repair‐deficient colorectal cancer (dMMR CRC), with limited data suggesting they may protect against recurrence. Our experimental study tested this hypothesis by investigating *B2M* mutation status and B2M protein expression and recurrence in patients in the stage II QUASAR clinical trial.

**Methods and results:**

Sanger sequencing was performed for the three coding exons of *B2M* on 121 dMMR and a subsample of 108 pMMR tumours; 52 with recurrence and 56 without. B2M protein expression was assessed by immunohistochemistry. Mutation status and protein expression were correlated with recurrence and compared to proficient mismatch repair (pMMR) CRCs. Deleterious *B2M* mutations were detected in 39 of 121 (32%) dMMR tumours. Five contained missense *B2M*‐variants of unknown significance, so were excluded from further analyses. With median follow‐up of 7.4 years, none of the 39 *B2M*‐mutant tumours recurred, compared with 14 of 77 (18%) *B2M‐*wild‐type tumours (*P* = 0.005); six at local and eight at distant sites. Sensitivity and specificity of IHC in detecting *B2M* mutations was 87 and 71%, respectively. Significantly (*P* < 0.0001) fewer (three of 104, 2.9%) of the 108 pMMR CRCs demonstrated deleterious *B2M* mutations. One pMMR tumour, containing a frameshift mutation, later recurred.

**Conclusion:**

*B2M* mutations were detected in nearly one‐third of dMMR cancers, none of which recurred. *B2M* mutation status has potential clinical utility as a prognostic biomarker in stage II dMMR CRC. The mechanism of protection against recurrence and whether this protection extends to stage III disease remains unclear.

## Introduction

The mismatch repair system (MMR) is responsible for recognising and repairing errors during DNA replication. Defects in this system lead to a hypermutation state and accumulation of genetic mutations. When this occurs within tumour suppressor genes or oncogenes, it predisposes to malignancy. Germline mutations in MMR genes give rise to Lynch syndrome, characterised by an inherited predisposition to early onset of tumours.^1^ Epigenetic silencing of the *MLH1* gene (promoter hypermethylation and associated *BRAF* mutation) also leads to mismatch repair‐deficient (dMMR) tumours, which represent approximately 15% of colorectal cancers (CRC).^2^ Two further proposed mechanisms account for a small proportion of Lynch syndrome cases; first, the presence of epimutations of *MLH1* and secondly the presence of heterozygous germline deletions of the 3 exons of the epithelial cell adhesion molecule (EPCAM). The former is characterised by promoter methylation and transcriptional silencing of a single allele of a gene in normal tissues, whereas in the latter, deletion of EPCAM results in transcriptional read‐through, thus silencing *MSH2*.^3^, ^4^

dMMR CRCs are biologically different from CRCs arising through the chromosomal instability pathway. They exhibit the characteristic histological appearances of the microsatellite instability phenotype (MSI‐H), including poor differentiation, mucinous histology and the presence of numerous tumour‐infiltrating lymphocytes.^5^ In stages II/III disease, dMMR tumours are associated with a favourable prognosis with improved survival compared to proficient MMR (pMMR) tumours.^6^, ^7^, ^8^, ^9^, ^10^ However, in stage IV CRC, dMMR is less frequent (3–5%) and is associated with a significantly worse prognosis.^11^ dMMR CRCs accumulate somatic mutations in genes which are usually highly conserved.

Beta2‐microglobulin (B2M) is a component of the human leucocyte antigen (HLA) class I complex, which is involved in the presentation of antigenic peptides, at the cell surface, to cytotoxic CD8^+^ T cells. The *B2M* gene contains several coding microsatellites, making it a mutagenic target in the presence of mismatch repair deficiency.^12^ Somatic *B2M* mutations are found in approximately 30% of dMMR CRCs, but are rare (<2%) in CRCs with proficient mismatch repair.^13^, ^14^, ^15^, ^16^, ^17^ In a consecutive series of MSI‐H CRCs, Kloor and colleagues^16^ detected *B2M* mutations in 29 of 104 (28%). All *B2M* mutations occurred within localised tumours (stages I–III) (23 of 68; 34%), but none occurred in nine CRCs with stage IV disease (*P* = 0.04). Kloor *et al.* 16 hypothesised that functional *B2M* is important in tumour development and *B2M* mutations may protect patients from developing distant metastases. These findings were replicated in a small cohort of MSI‐H CRCs (*n* = 34) from the FOGT‐4 trial. *B2M* mutations were identified in 10 of 34 tumours (29%), none of which recurred during 5 years follow‐up (none of 10), while six of 24 (25%) *B2M*‐wild‐type MSI‐H CRCs recurred (*P* = 0.09), all within 12 months of surgery.^12^ These studies suggested that *B2M* mutation status might provide useful prognostic information, but were inconclusive because of their small size.

The aim of this study was to determine the frequency of somatic *B2M* mutations in a sample of dMMR and pMMR CRC specimens from a large randomised controlled clinical trial (QUASAR) and correlate mutation status with B2M protein expression and recurrence.

## Materials and methods

In the QUASAR trial (ISRCTN82375386), patients with an uncertain indication for chemotherapy following curative CRC resection were randomised to receive 5‐fluorouracil/folinic acid or observation and followed‐up for a median of 5.5 years. A total of 3239 patients were recruited to the study (71% colon cancer; 29% rectal cancer), with the majority (91%) having stage II disease. Adjuvant chemotherapy was associated with better recurrence‐free and overall survival with an absolute survival advantage of 3.6% at 5 years.^18^

Tumour blocks and DNA samples were obtained retrospectively for CRCs from the QUASAR trial. Tumours had previously been characterised as dMMR, with loss of either MLH1 or MSH2 protein expression by immunohistochemistry.^8^ Hutchins and colleagues investigated the value of mismatch repair status in predicting recurrence in the QUASAR samples. Recurrence rates for the 218 of 1913 (11%) CRCs that had MLH1 or MSH2 loss (dMMR CRC) were 11% compared with 26% for pMMR tumours [risk ratio (RR) = 0.53; 95% confidence interval (CI) = 0.40–0.70; *P* < 0.001].^8^

### Beta2 Microglobulin Mutation Analysis

The study was approved by the South Manchester Research Ethics Committee (10/H1003/11). All three coding exons of the *B2M* gene were amplified in four 20‐µl polymerase chain reactions (PCR) using N13‐tailed forward and reverse primers (see Supporting information, Data S1). PCR was performed using a Veriti 96‐well thermal cycler. PCR products were purified prior to bi‐directional BigDye version 3.1 Sanger sequencing using Agencourt AMPure XP beads and following sequencing Agencourt CleanSeq beads with a Biomek NX Laboratory Automation Workstation (Beckman Coulter, High Wycombe, UK). Sanger sequencing reactions were analysed using a 3730 DNA Analyser (Applied Biosystems, Warrington, UK).

### Genotyping

Mutation analysis was performed using trace subtraction software (Staden package; www.sourceforge.net). Tumour sequence data were compared against sequence data from a normal control sample. Mutations and variants were named according to Human Genome Variation Society nomenclature using the *B2M* reference sequence NM_004048.2 (www.hgvs.org/mutnomen). All mutations and potentially deleterious variants were confirmed by sequencing of an independent PCR amplification. Frameshift and non‐sense mutations within coding regions of *B2M* exons and mutations affecting the invariant splice site (within two base pairs of the flanking intronic sequence) were considered significant. Synonymous mutations and intronic mutations were considered to be insignificant. Missense mutations were considered variants of uncertain significance and were analysed separately. The probable effect upon protein expression and function was predicted using software within Alamut version 2.2 (Interactive Biosoftware, Rouen, France). As almost all *B2M* mutations in CRC occur within exon 1 and the portion of exon 2 covered by the 2a amplicon,^19^ samples were included even if an analysable result could not be obtained for the 2b amplicon or exon 3.

### Immunohistochemical Analysis

**Figure 1:**
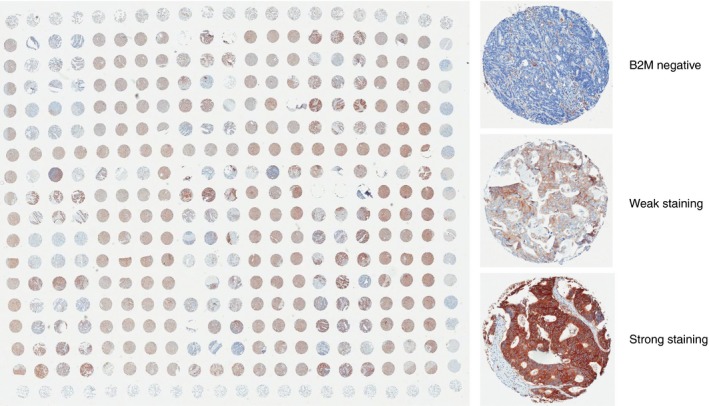
A tissue microarray (TMA) containing cores of tumour tissue from 42 colorectal cancers from the QUASAR study, with additional cores of sheep lung, liver, brain and placenta as orientation markers. Tissue scores were scored as ‘no staining’ (0), ‘weak staining’ (1) or ‘strong staining’ (2)

Tissue microarrays (TMA) containing cores from up to 42 cases each had previously been constructed. Three representative cores of tumour tissue plus three cores of tumour‐associated normal tissue were used per patient (Figure 1).

Immunohistochemistry (IHC) was performed on 5‐m sections from each TMA using the Dako EnVision + system (Dako, High Wycombe, UK). Slides were stained with primary antibody to B2M protein (rabbit polyclonal, NCL‐B2Mp; Leica Biosystems, Newcastle upon Tyne, UK) at 1:800 dilution and horseradish peroxidise (HRP)‐labelled anti‐rabbit secondary antibody. The immunological reaction was visualised using 3, 3‐diaminobenzidine (DAB) as chromogen and counterstained with haematoxylin. Slides were scanned at × 20 magnification and visualised using Aperio ImageScope software (version 11.1). Tumours were scored as no staining (0), weak staining (1) or strong staining (2) by two independent researchers (P.B., S.D.R.) taking an average score across the three cores, and disagreements were resolved by consensus (Figure 1). Positively stained stromal cells were used as an internal positive control.

### Statistical Analysis

A power calculation was based on a 28% *B2M* mutation frequency in dMMR CRC^16^ and 20% recurrence rate within QUASAR.^16^ At 5% significance level and 80% power, the number of tumours required to detect 0% and 20% recurrence rates in tumours with and without a *B2M* mutation, respectively, was 108. Mutation analysis was performed blinded to the clinical outcome and results reported to the QUASAR collaborative group. Correlation between *B2M* mutation status and IHC protein expression and recurrence was explored using Mantel–Haenszel tests for association and log‐rank time‐to‐event analyses. A probability of < 0.05 was taken to indicate statistical significance. For pMMR tumours, the *B2M* mutation frequency was predicted to be lower, and therefore only a subsample were tested in a case–control study enriched for recurrence. Equal numbers of recurrent and non‐recurrent tumours were selected at random from the QUASAR database.

## Results

### B2M Mutation Frequency

One hundred and forty‐seven dMMR CRC samples were analysed (144 colon, two rectum, one rectosigmoid); 26 samples failed to amplify or results could not be confirmed. Verified results were obtained for 121 samples (102 *MLH1* loss, 19 *MSH2* loss). Thirty‐nine of 121 (32.2%) contained pathogenic *B2M* mutations (see Supporting information, Data S2). Twenty‐one samples contained one pathogenic mutation and 18 samples carried more than one. Overall, 58 pathogenic mutations were detected in 39 samples. Eleven samples contained missense mutations of uncertain significance, six in conjunction with a pathogenic *B2M* mutation (see Supporting information, Data S3). There was no significant difference in *B2M* mutation frequency based on whether MLH1 or MSH2 protein expression had been lost (*P* = 0.46) or *BRAF* mutation status (*P* = 0.87).

Fifty‐five of 58 (95%) pathogenic *B2M* mutations occurred within exon 1 or exon 2 amplicon 2a, with the majority of these being frameshift mutations (47 of 58; 81.0%). Most frameshift mutations occurred at nucleotide repeats (36 of 47; 76.6%), with nearly half (23 of 47; 48.9%) occurring at a (CT)_4_ mutational ‘hot‐spot’ in exon 1. Fourteen different frameshift mutations were identified. The most frequent mutation was c.43_44delCT p.(Leu15PhefsTer41), which was detected in 23 samples.

Seven non‐sense mutations were identified which cause premature termination of the B2M protein. These occurred throughout all exons of the *B2M* gene and were classified as pathogenic. Three splice site mutations were identified which were predicted to significantly affect protein expression. One stop codon mutation was also detected [c.360A> C p.(Ter120TyrextTer49)].

### Correlation of B2M Mutations with B2M Protein Expression

B2M protein expression was available for 88 samples with known *B2M* mutation status. Protein expression was assessed independently by P.B. and S.D.R., with interobserver agreement of 83 of 88 (94%). Of the 23 tumours with pathogenic *B2M* mutations, 20 (87%) showed complete loss of B2M expression (sensitivity 87%). Thirteen of 23 (57%) contained two or more pathogenic *B2M* mutations and all but one of these 13 had complete loss of *B2M* expression (12 of 13; 92%). The samples with pathogenic *B2M* mutations and no loss of B2M expression on IHC had a frameshift mutation in exon 1 (one sample) and non‐sense mutations in exons 1 and 2a (two samples). Of 61 tumours with wild‐type *B2M*, 18 (30%) had complete loss of B2M expression on IHC (specificity: 70%). All four samples tested with isolated missense mutations maintained B2M expression.

### Effect of B2M Mutations and Protein Expression on Recurrence

**Figure 2:**
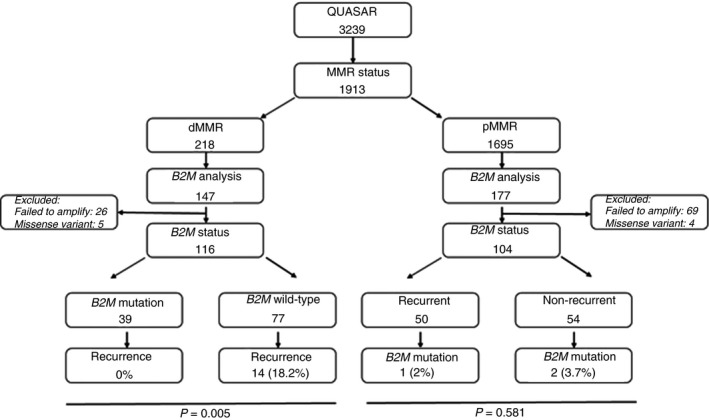
Consort diagram of samples in the QUASAR study and subsequent studies

Five of the 121 dMMR CRCs with isolated missense variants of uncertain significance were excluded, leaving 116 tumours for analysis (Figure 2). Thirty‐nine samples had a pathogenic *B2M* mutation and 77 had wild‐type *B2M*. With a median of 7.4 years of follow‐up, none of the 39 tumours with significant *B2M* mutations recurred compared to 14 of 77 (18.2%) of tumours with wild‐type *B2M* (*P* = 0.005).

B2M loss via epigenetic mechanisms did not appear to offer complete protection from recurrence. Three of 18 tumours with B2M protein loss in the presence of a wild‐type *B2M* gene recurred. In addition, of 26 tumours which failed to amplify, 10 tumours demonstrated protein loss by IHC, two of which recurred. Of interest, one of the tumours with an isolated missense variant recurred.

Of 177 pMMR CRCs tested, 69 failed to amplify so analysable results were available for 108 pMMR samples, 52 from recurrent tumours and 56 from non‐recurrent tumours. Four missense mutations of uncertain significance were identified (two recurrent, two non‐recurrent), and were excluded. Significantly fewer (*P* < 0.0001) of the pMMR than dMMR CRCs contained *B2M* mutations: 2.9% (three of 104) versus 36% (39 of 108). Two pMMR tumours had frameshift mutations (c.43_44delCT and c.204delA) and one a splice site mutation (c.68‐2A> G). The tumour containing the commonly found c.43_44delCT frameshift mutation recurred.

**Figure 3:**
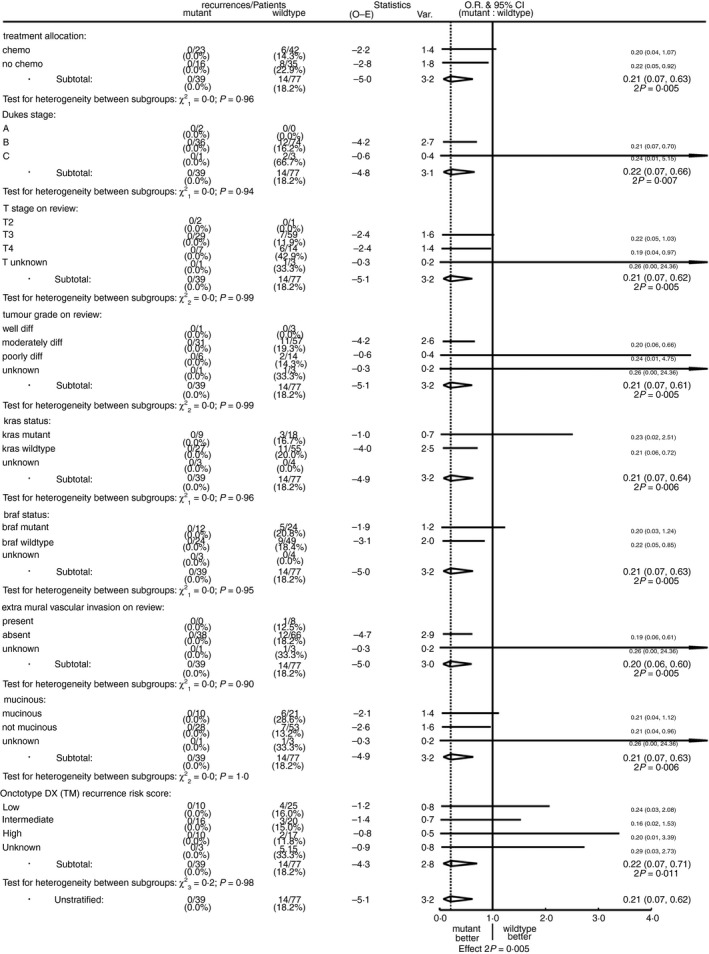
Recurrence by *B2M* status stratified by chemotherapy allocation, Dukes stage, T‐stage, tumour grade, *KRAS* status, *BRAF* status and oncotype DX risk score

*B2M* status was not significantly related to any tumour characteristics other than MMR status. Similar proportions of the *B2M*‐mutant and *B2M*‐wild‐type dMMR tumours were T4 stage [18% (seven of 38) versus 19% (14 of 74)], poorly differentiated [16% (six of 38) versus 19% (14 of 74)], mucinous [26% (10 of 38) versus 28% (21 of 74)], had *KRAS* mutations [25% (nine of 36) versus 25% (18 of 73)], *BRAF* mutations [33% (12 of 36) versus 28% (21 of 74)] and high oncotype DX recurrence scores [28% (10 of 36) versus 27% (17 of 62)]. The *B2M*‐mutant tumours were, however, less locally invasive with none exhibiting extramural invasion, significantly fewer than among the *B2M*‐wild‐type tumours: 0% (none of 38) versus 16% (12 of 74), *P* = 0.009. As 95% (110 of 116) of the tumours tested were stage II, consistent with the 91% overall proportion of stage II CRCs in QUASAR, we could not meaningfully compare propensity for nodal spread in *B2M*‐mutant and wild‐type tumours: 2.6% (one of 39) versus 3.9% (three of 77) were node‐positive. With no strong associations between *B2M* status and these other variables, comparisons of recurrence rates between *B2M*‐mutant and wild‐type tumours adjusting for other variables produced near identical results to the unstratified analyses (Figure 3).

**Figure 4:**
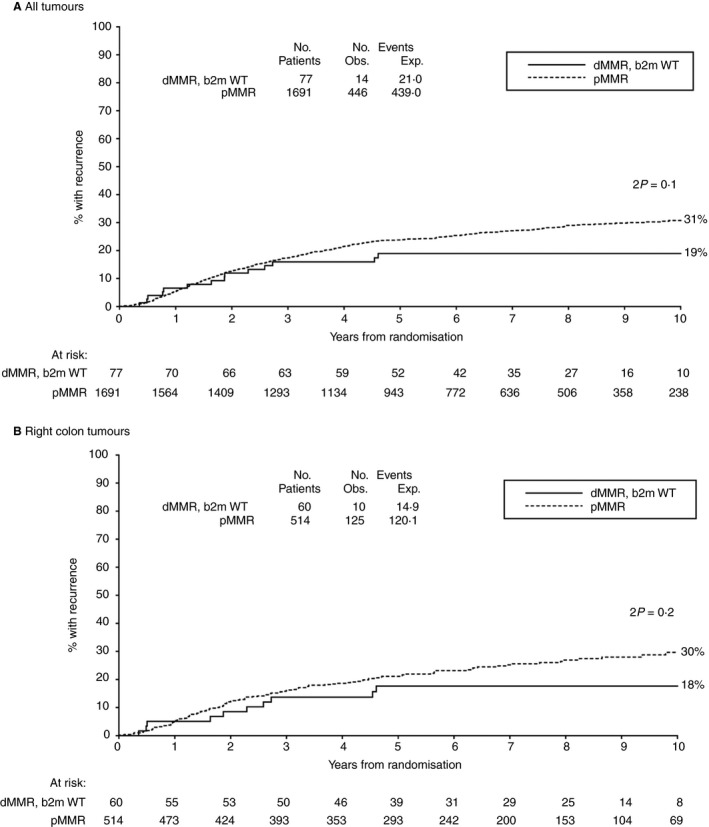
Ten‐year risk of recurrence for MMR‐deficient, *B2M*‐wild‐type tumours compared to MMR‐proficient tumours in (**A**) all patients and (**B**) right colon tumours only

**Figure 5:**
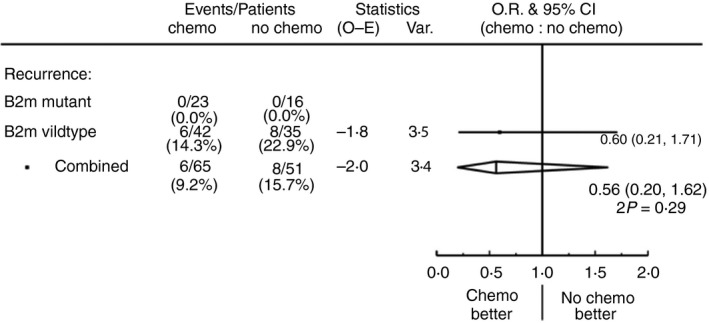
Recurrences by treatment allocation in MMR‐deficient tumours with and without *B2M* mutations

To assess whether the previously reported better prognosis of MMR‐deficient tumours^8^ might be explained by their association with *B2M* mutations, we compared the recurrence rate in dMMR *B2M*‐wild‐type tumours with that in all MMR‐proficient tumours in the QUASAR trial, and found no significant difference overall or in analyses restricted to right‐sided colon tumours, where most dMMR tumours arise (Figure 4). There were too few recurrences in the dMMR *B2M*‐wild‐type tumours to assess whether chemotherapy was equally effective in such tumours compared to pMMR tumours, but the trend towards fewer recurrences in the chemotherapy than control groups [14.3% (six of 42) versus 22.9% (eight of 35) recurred, risk ratio = 0.56; 95% CI = 0.20–1.62, 2*P* = 0.29] (Figure 5) is consistent with equal efficacy of chemotherapy in this subgroup.

The immune infiltration within each of the 39 B2M‐mutant dMMR was assessed by review of the haematoxylin and eosin (H&E)‐stained slides. An equally sized group of dMMR B2M‐wild‐type tumours was selected at random. Infiltration was graded as ‘0’, no infiltration; ‘1’, scattered inflammatory cells; ‘2’, very mild band‐like infiltrate with at least one aggregate; ‘3’, moderate band‐like infiltrate with follicles and ‘4’ dense band‐like infiltrate. Infiltration was then dichotomised into ‘low; ‘0–2’ and high; ‘3 and 4’. Within the B2M‐mutant group, 38 H&Es were assessable and of these, 30 (78.9%) showed low infiltration. Of the 43 assessable B2M‐wild‐type tumours, 79.1% (34 of 43) were deemed to show low infiltration, making the difference between the two groups non‐significant.

## Discussion

This study has determined *B2M* mutation status and its influence on recurrence in QUASAR, the largest randomised trial of adjuvant chemotherapy in colorectal cancer. Thirty‐nine of 121 (32%) of dMMR CRCs contained deleterious *B2M* mutations. We have shown that *B2M* mutations were strongly associated with absence of recurrence (none of 39), compared to 14 of 77 (18%) with wild‐type *B2M* over 10 years’ follow‐up, a highly statistically significant difference (*P* < 0.005).

Three other studies have reported an absence of recurrence in *B2M*‐mutant dMMR CRC (Table 1). Only one was prospective within the context of a randomised trial, but numbers were small (*n* = 34).^10^ Koelzer *et al.* 20 identified B2M loss in 19 of 98 (19.4%) dMMR CRC patients using IHC. There were no recurrences (none of 19) in patients with B2M loss, but 14 of 79 (17.7%) in those with maintained B2M expression. This was reflected in prolonged 5‐year survival (91.7% versus 72.1%)*.* 20 In combination with the current study, 91 dMMR tumours with *B2M* deficiency did not recur, seven of which were stage III tumours, compared with 43 of 234 (18.4%) with proficient *B2M* that did recur. A log‐rank test for the difference in numbers of recurrence in B2M mutation/B2M loss and B2M‐proficient, stratified by study, yields a χ^2^ of 19.01, *P* = 0.00001.

**Table 1:** Composite results of studies comparing outcome of dMMR CRC based on *B2M* mutation status

Study	Tumour stage	Recurrence/metastases	
		*B2M* mutation/B2M loss	*B2M*‐proficient
Kloor *et al.* (2007) Case–control	58% stages I/II 30% stage III 12% stage IV	0/23	9/54
Tikidzhieva *et al*. (2012) Randomised trial	24% stages I/II 76% stage III	0/10	6/24
Koelzer *et al*. (2012) Case–control	53% stages I/II 47% stage III	0/19	14/79
Current study Randomised trial	95% stage II 5% stage III	0/39	14/77
Total		0/91	43/234

dMMR, mismatch repair‐deficient; CRC, colorectal cancer.

As in previous studies, we found that *B2M* mutations are rare in pMMR CRC (< 3%), as it is the coding microsatellites in the *B2M* gene which are most vulnerable to mutation in the presence of dMMR.^13^, ^15^, ^17^ Koelzer and colleagues^20^ found a somewhat higher rate of B2M protein loss among pMMR tumours (22 of 310; 7%), although three (14%) of these 22 tumours recurred and no survival advantage was demonstrated. In our study, significant *B2M* mutations occurred in 2.9% (three of 104) pMMR CRCs, and the sample containing the most frequent frameshift mutation recurred. We acknowledge that a higher proportion of pMMR samples failed to amplify compared to dMMR (39% versus 17%), although the main reasons for this are technical. The DNA was extracted many years ago, using an in‐house phenol–chloroform extraction method, which we suspect to have contributed to the failure to amplify of so many samples. However, this was unlikely to have been influenced by MMR status.

We must acknowledge the limitations of determining mismatch repair status purely on the basis of only MLH1 or MSH2 expression. At the time of testing, MSH6 and PMS2 were not established as routine biomarkers. It is possible that a small number of cases, deemed pMMR on the basis of retained MLH1 and MSH2 expression, would actually have shown loss of one of MSH6 or PMS2 alone; however, this number would have been extremely low, and did not warrant restaining each case.

The use of TMAs allowed high throughput and cost‐effective immunohistochemical staining. We deemed that three 0.6‐mm cores, taken at random, were representative of each individual tumour. As staining across the three cores from each case was consistent, we decided that whole section staining was not required. Tissue from the QUASAR trial is a limited and valuable clinical trial resource, so minimising its use by staining the already‐made TMAs was thought to be the most sensible use of the tissue. We acknowledge that there were a number of cases where the B2M protein expression was not available. This was due primarily to the tissue cores on the TMA section either falling off the slide during the staining process or the core having been sectioned through so often that there was no longer sufficient tumour tissue remaining to be analysed.

Sensitivity of IHC for significant *B2M* mutations was 86.9% and specificity was 70.5%. Factors accounting for this discrepancy may include the persistent expression of B2M epitope in the presence of a non‐sense mutation, particularly if the mutation is in the distal part of the gene. Conversely, loss of staining in tumours with wild‐type *B2M* might be due to large genomic rearrangements not identifiable with Sanger sequencing, promoter mutations, mutations involving miRNA recognition sites, low neoplastic cell content (<20%) or sampling error. In our study, B2M loss via epigenetic mechanisms did not confer complete protection from recurrence, as we identified three tumours with B2M protein loss in the presence of wild‐type *B2M* which recurred. Intratumour heterogeneity remains a considerable challenge, and it may be that combining DNA from multiple FFPE tumour blocks would be more effective for *B2M* testing, as demonstrated for *KRAS.* 21 Deeper and more expansive ‘next‐generation sequencing’ approaches might also identify a higher proportion of *B2M* mutations among dMMR CRCs, but results with formalin‐fixed paraffin‐embedded (FFPE) specimens have been variable and would require careful validation.

### B2M Mutations, Loss of HLA class I and Recurrence

The mechanism of protection from distant metastases afforded by somatic *B2M* mutations is yet to be fully elucidated. B2M forms an essential part of the HLA class I complex on the surface of all nucleated cells. Deleterious *B2M* mutations lead to loss of HLA class I expression, which allows tumour cells to avoid recognition by cytotoxic T lymphocytes but activates natural killer (NK) cells to destroy tumour cells in circulation, as a result of missing ‘self‐recognition’.^13^, ^15^, ^17^, ^22^ There is growing evidence that HLA class I‐deficient tumour cells are less likely to establish distant metastases than HLA class I‐proficient cells. Menon and colleagues^23^ showed that loss of HLA class I expression is rare in colorectal metastases, and concordance of HLA expression between primary and metastatic lesions is high. In uveal melanoma, which has many similarities with dMMR CRC (preferential haematogenous spread to the liver and similar *B2M* mutation pattern), loss of HLA‐class I/B2M expression is also associated with significantly improved survival.^24^, ^25^ The role of NK activity and HLA class I loss in the prevention of distant metastases has been extensively investigated in uveal melanoma and fibrosarcoma, in both in *in‐vitro* studies and mouse models.^26^, ^27^, ^28^, ^29^

Kloor *et al.* 16 suggested that *B2M* mutations might enable the tumour cells to evade local immune responses and promote local tumour growth. In our study, of the 14 recurrences in dMMR *B2M* wild‐type tumours, six were local, six distant and two both local and distant. *B2M* mutations appear to protect against local as well as distant recurrence, and this was separately significant (none of 39 versus eight of 77; *P* = 0.038).

The protective effect of *B2M* mutations appears to be limited to dMMR. The presence of hypermutation stimulates an augmented immune response, as seen from histopathological examination of dMMR tumours. In *B2M*‐mutant dMMR tumours, the immune response is unleashed in the presence of abnormal class I presentation, but in pMMR the immune response is less marked, possibly leading to survival of the metastatic clones. We propose that *B2M* testing is a useful adjunct to routine MMR testing and should be incorporated into a bowel tumour‐specific assay in conjunction with *MLH1*, *KRAS*, *NRAS* and *BRAF* status to provide an overall recurrence risk for individual patients.

We have tested the validity of *B2M* mutation as a prognostic biomarker of recurrence in dMMR CRC in a large prospective randomised clinical trial. Results indicate that patients with dMMR CRC who have a *B2M* mutation are protected from developing recurrent disease following resection. *B2M* status is a more accurate prognostic marker than MMR status alone, and the high prevalence of *B2M* mutations in dMMR disease may well explain the better prognosis of dMMR compared to pMMR CRC. Approximately 15% of all CRC and virtually all patients with Lynch syndrome cancers are dMMR, and one‐third of these will also have a somatic *B2M* mutation. The one patient with stage III disease in our study did not recur, nor did six other patients with stage III *B2M*‐mutant tumours in the only other prospective study.^12^ If patients with *B2M*‐mutant stage III dMMR CRC are also protected from recurrence, *B2M* mutation status would have additional clinical utility.

## Conflicts of interest

The authors have declared no conflicts of interest.

## Supporting information


**Data S1** **.** Forward and reverse primer sequences used for *B2M* PCR reaction. Primers were designed to avoid polymorphisms and Alu repeats using Primer 3 (http://primer3.ut.ee/ and SNPCheck3 (http://secure.ngrl.org.uk/SNPcheck). PCR primers were tagged with an ‘N13’ universal sequencing tail (Forward: 5′‐GTAGCGCGACGGCCAGT; Reverse: 5′‐CAGGGCGCAGCGATGAC). Primers were obtained from Sigma‐Aldrich**^®^**. Thermal cycling conditions were initial denaturation 2 mins at 96°, denaturation 30 cycles of 10 secs at 96°, annealing 20 secs at 55°, extension 4 mins at 60° and hold at 15°.

**Data S2** **.** Description and frequency of *B2M* mutations identified in the QUASAR dMMR CRC samples.

**Data S3** **.** Description, frequency and distribution of missense *B2M* mutations and predicted *in silico* effect of protein structure and function (*occurred in conjunction with a pathogenic mutation).
