# Supplementary material for: Confirmation that somatic mutations of beta‐2 microglobulin correlate with a lack of recurrence in a subset of stage II mismatch repair deficient colorectal cancers from the QUASAR trial
Source: Histopathology. 2019 Jul 5;75(2):236–46. doi: 10.1111/his.13895 (PMC6772160; doi:10.1111/his.13895)
Supplement: Supplementary file 1 — Data S1 . Forward and reverse primer sequences used for B2M PCR reaction. Primers were designed to avoid polymorphisms and Alu repeats using Primer 3 (http://primer3.ut.ee/ and SNPCheck3 (http://secure.ngrl.org.uk/SNPcheck). PCR primers were tagged with an ‘N13’ universal sequencing tail (Forward: 5′‐GTAGCGCGACGGCCAGT; Reverse: 5′‐CAGGGCGCAGCGATGAC). Primers were obtained from Sigma‐Aldrich®. Thermal cycling conditions were initial denaturation 2 mins at 96°, denaturation 30 cycles of 10 secs at 96°, annealing 20 secs at 55°, extension 4 mins at 60° and hold at 15°. [file HIS-75-236-s001.docx]

**S1:** **ON-LINE ONLY**. Forward and reverse primer sequences used for *B2M* PCR reaction. Primers were designed to avoid polymorphisms and Alu repeats using Primer 3 (http://primer 3.ut.ee/ and SNPCheck3 (http://secure.ngrl.org.uk/SNPcheck). PCR primers were tagged with an ‘N13’ universal sequencing tail (Forward: 5’-GTAGCGCGACGGCCAGT; Reverse: 5’-CAGGGCGCAGCGATGAC). Primers were obtained from Sigma-Aldrich**^®^**. Thermal cycling conditions were initial denaturation 2 mins at 96^o^, denaturation 30 cycles of 10 secs at 96^o^, annealing 20 secs at 55^o^, extension 4 mins at 60^o^ and hold at 15^o^.

| **Exon** | **Primers** | **Size** |
| --- | --- | --- |
| Exon 1 | Forward: 5’-CCCTCTCTCTAACCTGGCACTG-3’  Reverse: 5’-ACGGAGCGAGAGAGCACAG-3’ | 299bp |
| Exon 2a | Forward: 5’-CACCAAGTTAGCCCCAAGTGA-3’  Reverse: 5’-AACTATCTTGGGCTGTGACAAAGT-3’ | 338bp |
| Exon 2b | Forward: 5’-GAGTATGCCTGCCGTGTGAA-3’  Reverse: 5’-TGGGATGGGACTCATTCAGG-3’ | 175bp |
| Exon 3 | Forward: 5’-GCTTGTTCCTGCTGGGTAGC-3’  Reverse: 5’-CCTCAGGACAGTGAAACAAAAACA-3’ | 237bp |
