# Supplementary material for: Confirmation that somatic mutations of beta‐2 microglobulin correlate with a lack of recurrence in a subset of stage II mismatch repair deficient colorectal cancers from the QUASAR trial
Source: Histopathology. 2019 Jul 5;75(2):236–46. doi: 10.1111/his.13895 (PMC6772160; doi:10.1111/his.13895)
Supplement: Supplementary file 2 — Data S2 . Description and frequency of B2M mutations identified in the QUASAR dMMR CRC samples. [file HIS-75-236-s002.docx]

| Exon | Nucleotide change | Amino acid change | Frequency |
| --- | --- | --- | --- |
| *Frameshift mutations: (n=47)* | | | |
| 1 | c.43_44delCT | p.(Leu15PhefsTer41) | 23 |
| 1 | c.45_48delTTCT | p.(Ser16AlafsTer27) | 1 |
| 2a | c.74_75delCA | p.(Pro25GlnfsTer31) | 1 |
| 2a | c.76_98dup23 | p.(His33GlnfsTer19) | 1 |
| 2a | c.125dupT | p.(Leu43ProfsTer14) | 1 |
| 2a | c.137_138delAT | p.(Tyr46CysfsTer10) | 1 |
| 2a | c.192_195delGAGA | p.(Arg65LeufsTer37) | 1 |
| 2a | c.194_195delGA | p.(Arg65AsnfsTer2) | 1 |
| 2a | c.204delA | p.(Val68TrpfsTer34) | 7 |
| 2a | c.204dupA | p.(Val69SerfsTer21) | 1 |
| 2a | c.231_306dup76 | p.(Asn103GlnfsTer12) | 1 |
| 2a | c.252_253delCT | p.(Leu85ValfsTer4) | 3 |
| 2a | c.276delC | p.(Thr93LeufsTer10) | 2 |
| 2a | c.276dupC | p.(Thr93HisfsTer2) | 3 |
|  |  |  |  |
| *Nonsense mutations: (n=7)* | | | |
| 1 | c.20T>A | p.(Leu7Ter) | 1 |
| 1 | c.64C>T | p.(Gln22Ter) | 1 |
| 2a | c.135C>A | p.(Cys45Ter) | 2 |
| 2a | c.240G>A | p.(Trp80Ter) | 1 |
| 2b | c.325C>T | p.(Gln109Ter) | 1 |
| 3 | c.349C>T | p.(Arg117Ter) | 1 |
|  |  |  |  |
| *Splice site mutations: (n=3)* | | | |
| 1 | c.67+2T>C | - | 1 |
| 2a | c.68-2A>G | - | 2 |
|  |  |  |  |
| *Initiation/stop codon mutation: (n=1)* | | | |
| 3 | c.360A>C | p.(Ter120TyrextTer49) | 1 |
|  |  |  |  |
| *Missense mutations: (n=11)* | | | |
| 1 | c.14T>C | p.(Val5Ala) | 1 |
| 1 | c.35T>C | p.(Leu12Pro) | 1 |
| 1 | c.37C>T | p.(Leu13Phe) | 1 |
| 1 | c.38T>C | p.(Leu13Pro) | 1 |
| 1 | c.38T>G | p.(Leu13Arg) | 1 |
| 2a | c.133T>C | p.(Cys45Arg) | 1 |
| 2a | c.152A>G | p.(His51Arg) | 1 |
| 2a | c.235G>A | p.(Asp79Asn) | 1 |
| 2a | c.274C>T | p.(Pro92Ser) | 1 |
| 2a | c.293A>G | p.(Tyr98Cys) | 1 |
| 2a | c.299G>C | p.(Cys100Ser) | 1 |

**S2:** **ON-LINE ONLY**. Description and frequency of *B2M* mutations identified in the QUASAR dMMR CRC samples.
