# Supplementary material for: Confirmation that somatic mutations of beta‐2 microglobulin correlate with a lack of recurrence in a subset of stage II mismatch repair deficient colorectal cancers from the QUASAR trial
Source: Histopathology. 2019 Jul 5;75(2):236–46. doi: 10.1111/his.13895 (PMC6772160; doi:10.1111/his.13895)
Supplement: Supplementary file 3 — Data S3 . Description, frequency and distribution of missense B2M mutations and predicted in silico effect of protein structure and function (*occurred in conjunction with a pathogenic mutation). [file HIS-75-236-s003.docx]

**S3:** **ON-LINE ONLY**. Description, frequency and distribution of missense *B2M* mutations and predicted *in silico* effect of protein structure and function (*occurred in conjunction with a pathogenic mutation).

| **Exon** | **Genomic change** | **Amino acid change** | **Frequency** | **Effect on protein structure/function** |
| --- | --- | --- | --- | --- |
| 1 | c.14T>C | p.(Val5Ala) | 1 | Unlikely |
| 1 | c.35T>C | p.(Leu12Pro) | 1* | Uncertain |
| 1 | c.37C>T | p.(Leu13Phe) | 1 | Unlikely |
| 1 | c.38T>C | p.(Leu13Pro) | 1* | Uncertain |
| 1 | c.38T>G | p.(Leu13Arg) | 1* | Uncertain |
| 2a | c.133T>C | p.(Cys45Arg) | 1* | Likely |
| 2a | c.152A>G | p.(His51Arg) | 1 | Likely |
| 2a | c.235G>A | p.(Asp79Asn) | 1* | Likely |
| 2a | c.274C>T | p.(Pro92Ser) | 1 | Likely |
| 2a | c.293A>G | p.(Tyr98Cys) | 1* | Likely |
| 2a | c.299G>C | p.(Cys100Ser) | 1 | Likely |
